# Reevaluation of the Dentary Structures of Caenagnathid Oviraptorosaurs (Dinosauria, Theropoda)

**DOI:** 10.1038/s41598-017-18703-1

**Published:** 2018-01-10

**Authors:** Shuo Wang, Qiyue Zhang, Rui Yang

**Affiliations:** 1 Laboratory of Vertebrate Evolution, College of Life Science, Capital Normal University, Beijing, 100048 China; 2 State Key Laboratory of Palaeobiology and Stratigraphy, Nanjing Institute of Geology and Palaeontology, Chinese Academy of Sciences, Nanjing, 210008 China

## Abstract

Among the characters of caenagnathid oviraptorosaurians, the lateral occlusal grooves and ridges on the occlusal surface of the jaw bones often receive special attention. Recent studies demonstrated that ontogenetic edentulism is present in caenagnathids, and therefore the lateral occlusal grooves and ridges are vestigial alveoli and interdental septa, respectively. In the present paper, the dentary structures of caenagnathids were reevaluated based on CT images of *Caenagnathiasia* sp. IVPP V20377. Several previously unknown features including crateriform vestigial alveoli, the morphology of the dentary interior hollow space, and the paired blind tubes beneath the dentary symphyseal shelf are recognized. Current lines of evidence suggest different jaw bone morphologies are likely produced by various tooth reduction patterns, which indicates ontogenetic dietary shift, if once presented in caenagnathids and *Sapeornis*, may have been different from the condition seen in *Limusaurus*. The 3D images of dentary interior spaces suggest that while tooth reduction progresses, the empty alveoli are partially modified into structures accommodating blood vessels that nourish the rhamphotheca, probably representing a functional compensation for the insufficient blood supply in toothed jaw bones.

## Introduction

Caenagnathidae is a monophyletic clade of oviraptorosaurian theropods known from the Upper Cretaceous of North American and Asia^1–5^, which is characterized by fully fused edentulous dentaries, a deep fossa on the lateral surface of the dentary, a ventrally extended preacetabular process of the ilium, and a medial fossa on the ischial peduncle of the pubis, among other characters^1,2^. Currently, most caenagnathids are described primarily based on fragmentary symphyseal regions of fused dentaries^1,3–9^ and dissociated postcranial elements^10–14^; well preserved articulated and semi-articulated skeletons are rare^2,15,16^. Caenagnathids exhibit a much greater range of body sizes than did other oviraptorosaurs^2^, from the ~8 m taxon *Gigantoraptor* ^15^ to *Caenagnathasia*, which is suggested to be less than 1 m in total length^3^. This suggests that their body sizes underwent a significant differentiation during the Late Cretaceous. Despite the range of body sizes, caenagnathids are also well known for a series of distinct dentary features including the hourglass-shape or dumbbell-shape depression on the ventral surface, the pneumatized dentary symphyseal region and rami, and the anterior and lateral grooves present on the occlusal surface of the dentary, making the caenagnathid dentary unique among theropod dinosaurs.

Distinct lateral occlusal grooves on the occlusal surface of the dentary separated from each other by the lateral occlusal ridges have been noticed for quite a long time. Sternberg^7^ skeptically suggested that these structures could be alveolar vestiges when he first described *Caenagnathus collinsi* some 80 years ago, while Currie *et al*.^5^ later interpreted them as structural support for the rhamphotheca, possibly with tomial tooth-like structures. Although several authors have mentioned these structures^1–4,6,8^, there is no consensus on their nature and function. A recent study has provided solid evidence suggesting the lateral occlusal grooves and ridges are vestigial alveoli and interdental septa, respectively^17^. Although this supports Sternberg’s suspicion, the possible function and significance of these vestigial periodontal tissues remain unclear. In the present paper, we re-describe a small specimen of *Caenagnathasia* sp. (IVPP (Institute of Vertebrate Paleontology and Paleoanthropology, Chinese Academy of Sciences) V20377)^3^, and investigate its internal morphology using CT scan and 3D reconstruction techniques. The available evidence suggests the vestigial alveoli have been partially modified into structures accommodating blood vessels that nourished the keratinized rhamphotheca after tooth loss, which broadens our understanding of the peramorphic development of the beaks in theropod dinosaurs^17,18^.

## Results

**Systematic Paleontology**

**Dinosauria Owen**, **1842**

**Saurischia Seeley**, **1887**

**Theropoda Marsh**, **1881**

**Oviraptorosauria Barsbold**, **1976**

**Caenagnathidae Sternberg**, **1940**

***Caenagnathasia*** **Currie**, **Godfrey & Nessov**, **1993**

***Caenagnathasia*** **sp**.

**Type species-** *Caenagnathasia martinsoni*, CCMGE (Chernshev’s Central Museum of Geological Exploration) 401/12457, completely fused dentaries^5^.

**Type locality and horizon-** Central Kyzylkum Desert, Uzbekistan, lower part of the Bissekty Formation (Upper Turonian)^5^.

**Referred specimen described in this study-** IVPP V20377, fused dentary preserved the symphyseal region and the anteriormost part of the rami^3^.

### Description

**Figure 1:**
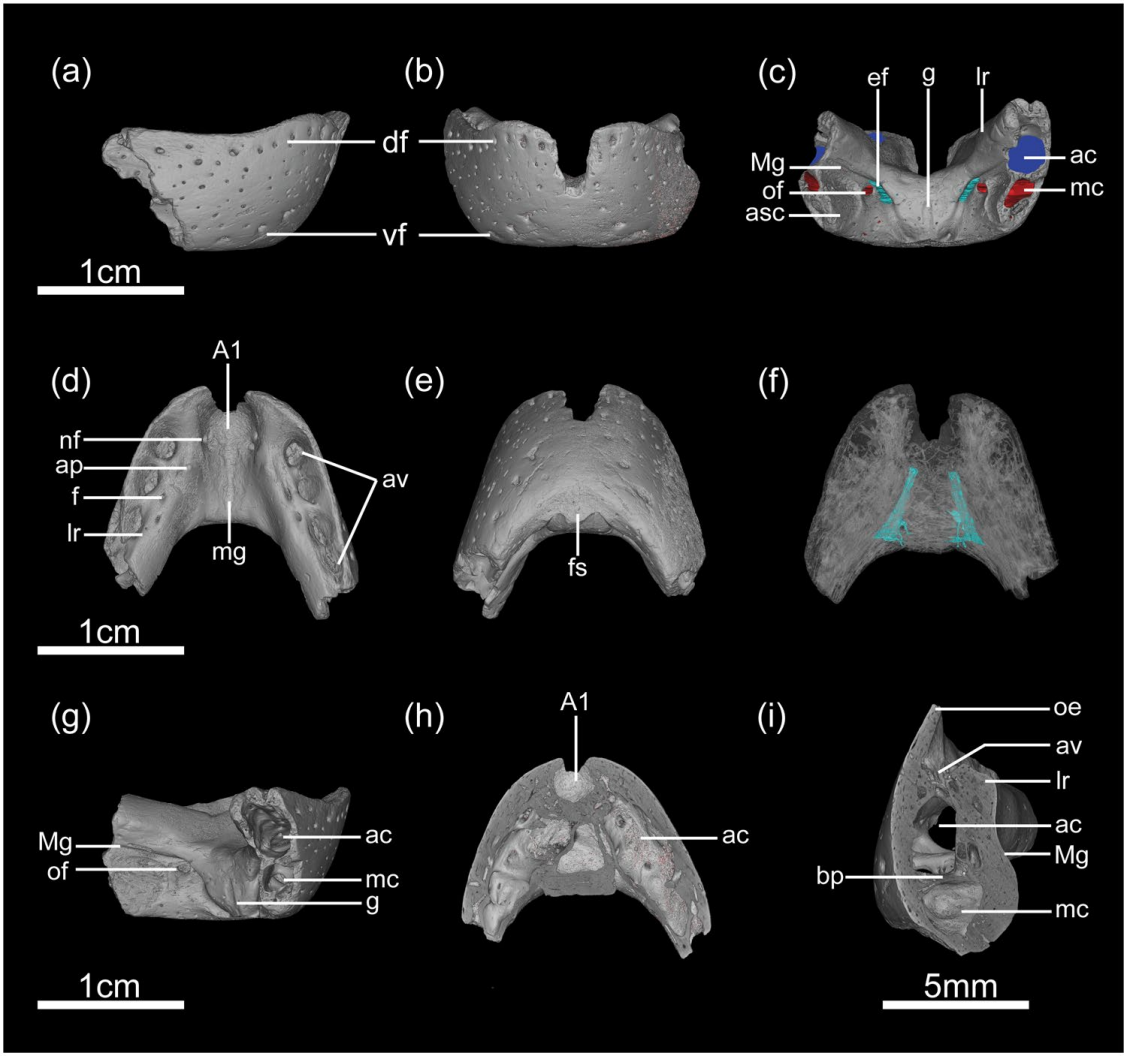
3D reconstruction of *Caenagnathasia* sp. IVPP V20377. (**a**) Right lateral view; (**b**) anterior view; (**c**) posterior view showing the alveolar canal (dark blue), mandibular canal (red), and the blind tubes open on the posterior surface of the symphyseal shelf (light blue); (**d**) dorsal view; (**e**) ventral view; (**f**) dorsal view showing the blind tubes (light blue) probably accommodating the anterior extension of Mecklian cartilage; (**g**) right posterolateral view; (**h**) horizontal section of the dentary showing the internal wall of the vestigial alveoli; (**i**) coronal section of the left dentary. Abbreviations: A1, anterior occlusal groove; ac, alveolar canal; ap, apical projection; asc, articular surface for the articular-surangular-coronoid complex; av, vestigial alveoli; bp, bony partition; df, dorsal row of foramina; ef, elongate foramen; f, foramen; g, groove; lr, lingual ridge; mc, mandibular canal; Mg, Mecklian groove; mg, midline groove; nf, neurovascular foramina; oe, occlusal edge; of, oval foramen; vf, ventral row of foramina. Scale bar for (**a**–**h**) = 1 cm, for (**i**) = 5 mm.

IVPP V20377 is a relatively small pair of fused dentaries that only preserves the symphyseal region and the anteriormost part of the ramus on each side (Fig. 1). The symphyseal shelf of this specimen is anteroposteriorly short, similar to that in *Chirostenotes* and *Leptorhynchos* ^1^, and is different from the anteroposteriorly elongate condition seen in *Caenagnathus* ^1,5,7^. The dentaries are completely fused together anteriorly as in all known caenagnathids except the perinate *Beibeilong* ^19^, in which they remain unfused, similar to the immature condition seen in oviraptorid embryos^20^. The contact of the dentaries is strengthened by the distinct symphyseal shelf and there is no trace of an intervening suture (Fig. 1).

**Figure 2:**
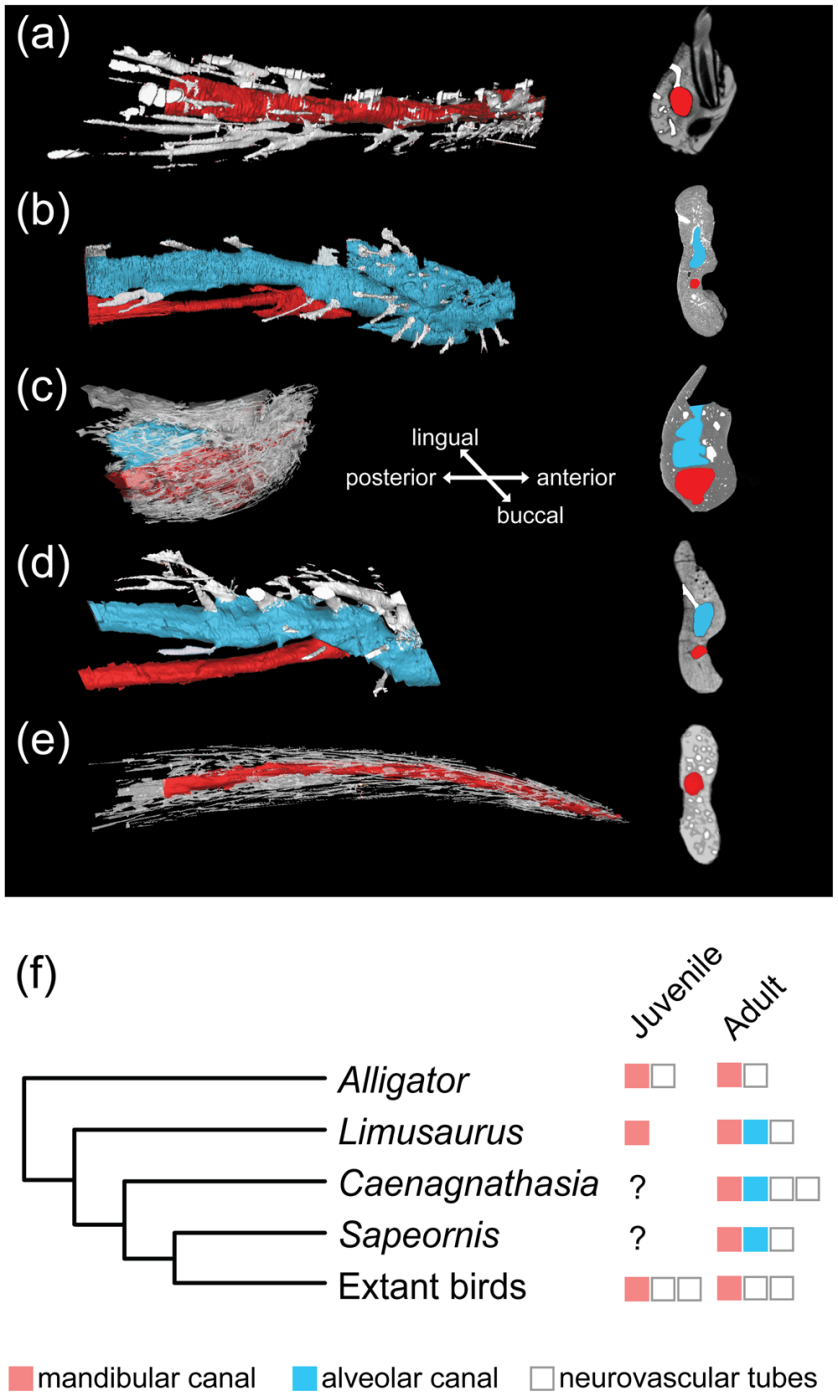
3D images of the denary interior spaces. (**a**–**e**) 3D molds of dentary interior spaces of (**a**) extant juvenile *Alligator sinensis* (IVPP 1361); (**b**) subadult *Limusaurus inextricabilis* (IVPP V15923); (**c**) *Caenagnathasia* sp. (IVPP V20377); (**d**) *Sapeornis chaoyangensis* and (**e**) extant *Pavo* sp. (IVPP 1032), showing the presence of the dentary mandibular canal (red), alveolar canal (blue) and neurovascular tubes (white) in both lateral (left) and coronal views (right); not to scale; (**f**) evidence from selected lineages showing the presence or absence of the vestigial alveoli, and the enrichment of neurovascular tubes.

In lateral view, the dentary forms a prominent sharp occlusal edge that is slightly concave, whereas the ventral margin is generally straight (Fig. 1a). The anterior margin of the dentary is only slightly convex, and it smoothly curves towarding the ventral margin without forming a chin-like eminence as in other caenagnathids^5,6,9^. The lateral surface of the dentary is generally smooth except for two linear series of large foramina that extend roughly parallel to the dorsal and ventral margins of the bone, respectively (Fig. 1a). These two rows of foramina converge anteriorly at the anterolateral corner of the sharp occlusal edge, and diverge as they extend posteriorly. Several smaller, irregularly arranged foramina are also present in the area bound by the linearly arranged foramina, but the area along the symphyseal midline is smooth and free of foramina as in other caenagnathids (Fig. 1b)^5^. CT images show that all foramina present on the outer surface of the dentary originate from the hollow interior space of the bone (Figs 1f and 2c), which would have carried capillaries and nerves that nourished the keratinized rhamphotheca as in other caenagnathids^5,8^. In some caenagnathids, such as *Anzu* ^2^, *Apatoraptor* ^16^, *Gigantoraptor* ^15^, *Leptorhynchos* ^1^ and *C*. *martinsoni* CCMGE 402/12457^5^, the lateral surface of the dentary anterior to the external mandibular fenestra is perforated by one or two pneumatopores passing anteromedially into the hollow interior space of the bone. It is difficult to tell whether a pneumatopore is also present in IVPP V20377 given most of its dentary rami are missing. In giant caenagnathids such as *Gigantoraptor* ^15^, *Anzu* ^2^ and MPC-D (Mongolian Paleontological Center, Mongolian Academy of Science) 107/17^8^, there is a prominent lateral flange present on the lateral surface of the dentary. This flange is very likely to have been absent from IVPP V20377 given that none of the known small caenagnathids possess this feature.

In dorsal view, although the anterior portion of the edge was broken off (Fig. 1b), the fused dentaries curve anteriorly toward the midline, giving the sharp occlusal edge a U-shaped appearance as in all known oviraptorosaurs^1,3,6,21,22^. The trough-like dorsal surface of the symphysis forms a large and deep oval-shaped anterior occlusal groove as in *C*. *martinsoni* ^5,6^. The floor of the anterior occlusal groove is not smooth, and its right lateral wall is pierced by two closely situated neurovascular foramina while the left wall is penetrated by a single foramen (Fig. 1d). This demonstrates that the blood vessels and nerves beneath this region are not strictly bilaterally distributed. CT images show the region underneath the anterior occlusal groove is filled with cancellous bone, whereas the foramina present in the anterior occlusal groove connect internally with the hollow space of the dentary through numerous thin sinuous tubes (Figs 1f and 2c). In other *C*. *martinsoni* (e.g. CCMGE 401/12457^5^ and ZIN (Zoological Institute, Russian Academy of Sciences) PH 2354/16^6^), the floor of the anterior occlusal groove is pierced by a single foramen on each side, but the foramen is located on the posterolateral corner of the groove, unlike those in IVPP V20377 that are on the lateral wall. In IVPP V20377, there is no sign of a second anterior occlusal groove lateral to the first anterior occlusal groove, a feature that contrasts with the condition seen in ZIN PH 2354/16^6^, but is similar to CCMGE 401/12457^5^.

In *Chirostenotes*, there is a distinct small tubercle present at the midpoint of the posterior margin of the anterior occlusal groove^5^. However, this tubercle is absent in all other small caenagnathid oviraptorosaurians including IVPP V20377, and in the large caenagnathids *Anzu* and *Gigantoraptor* ^2,15^, probably representing an autapomorphy of *Chirostenotes*. The symphyseal shelf posterior to the anterior occlusal groove is a shallow trough-like region, which is laterally bordered on each side by a swollen lingual ridge. This region is much broader transversely and shallower than the anterior occlusal groove, with an indistinct groove that extends along the midline (Fig. 1d). As previously described^3^, this midline groove is continuous anteriorly with the anterior occlusal groove as in *C*. *martinsoni* CCMGE 401/12457^5,6^, though these detailed features are vague and only faintly visible in CT images (Fig. 1d). In IVPP V20377, this midline groove has a rough surface and is even in depth, whereas it becomes deeper as it extends posteriorly in *C*. *martinsoni* CCMGE 401/12457^6^, representing a difference between these two specimens. Lateral to the midline groove, the dorsal surface of the symphyseal region is fairly flat without bearing any sign of longitudinal vascular grooves.

The two lingual ridges converge toward the midline as they extend anteriorly, and abruptly straighten at the posterior margin of the anterior occlusal groove (Fig. 1d), unlike *Caenagnathus* in which the lingual ridge from both sides join into a single ridge at the trough-like region of the symphyseal shelf^5,7,23^. Anterior to the inflection point, the lingual ridge steeply ascends as it extends anteriorly to merge with the lingual aspect of the occlusal edge (Fig. 1d). Posterior to the inflection point, the lingual ridge only gradually ascends as it extends posteriorly, and finally becomes as high as the occlusal edge at a position posterior to the fourth lateral occlusal groove (Fig. 1d). Thus the lingual ridge is much lower than the sharp occlusal edge at the inflection point when viewed dorsolaterally. Immediately posterior to the inflection point, each lingual ridge bears a small but distinct apical projection as in *C*. *martinsoni* CCMGE 401/12457 (Fig. 1d)^6^. However, this projection is clearly absent in ZIN PH 2354/16^6^. It should be noted that a similar projection is present in *Chirostenotes* TMP (Royal Tyrrell Museum of Paleontology) 2001.12.12^9^, though the lingual ridges do not extend as far anteriorly as those in IVPP V20377. Posterior to this apical projection, the lingual ridge is dorsally pierced by small oval foramina (Fig. 1d), and each is connected to the hollow interior space through a narrow tube (Fig. 1f). Previous study indicated that there are only two foramina present on each lingual ridge^3^, one located medial to the second vestigial alveolus and another medial to the third vestigial alveolus. However, CT images reveal an additional tiny foramen on the left side, which is immediately medial to the first vestigial alveolus (Fig. 1d), indicating that the branches of capillaries in this area are not bilaterally distributed.

Together, the lingual ridge and the sharp occlusal edge border the lingual groove on each dentary (Fig. 1d), which contains a series of vestigial alveoli that have been previously referred to as the “lateral occlusal grooves”^1,3–8^. A recent study suggested that there are three and five vestigial alveoli present in the left and right dentary, respectively^3^, but careful observation confirms that the right dentary has only four alveoli (Fig. 1d). The most anterior alveolus is separated from the anterior occlusal groove by the lingual ridge, whereas the rest of the alveoli are separated from one another by the vestigial interdental septa, which have been previously referred to the “lateral occlusal ridge” by some authors^5^. The vestigial interdental septa extend across the lingual groove to contact the lingual ridge, unlike the condition in most other caenagnathids (e.g. *Caenagnathus collinsi* CMN (Canadian Museum of Nature) 8776^5^) in which the lateral occlusal ridges fail to reach the lingual ridge. All vestigial alveoli are crateriform, meaning the opening is larger than the cavity (Fig. 1d,h). The first alveolus on each side is nearly circular, whereas the second and the third are slightly compressed transversely and smaller than the first one (Fig. 1d). The fourth alveolus on the right side, the smallest alveolus in this specimen, is significantly reduced and compressed transversely (Fig. 1d).

CT data show that the inner surfaces of the vestigial alveoli are excavated by tiny, irregularly distributed fossae (Fig. 1g), and that some vestigial interdental septa are rod-like and do not extend downward to completely separate the alveoli (Fig. 1h,i). The bottom of the alveolus is not enclosed as indicated by previously authors^5^, and it is penetrated by tiny foramina that communicate with the hollow space beneath (Fig. 1h,i). The hollow space inside the bone, as revealed by CT images, is very complex (Figs 1f and 2c). The breakage of the dentary ramus reveals two internal longitudinal canals, one located above the other (Fig. 1c,g,h,i). Our previous work has shown that the ventral canal is the mandibular canal, carrying the mandibular nerve (V_3_) and interior alveolar artery and vein in all vertebrates, whereas the dorsal canal is homologous to the vestigial alveoli^17^. CT images confirm that both canals span much of the length of the dentary and connect anteriorly in the symphyseal region, with the mandibular canal separated from the alveolar canal by a discontinuous horizontal bony plate (Figs 1c,g,h,i and 2c), reminiscent of the condition present in edentulous adult *Limusaurus* ^18^. 3D images of the dentary interior spaces also indicate that some of the neurovascular foramina present on the lateral surface of the dentary connect with the alveolar canal (Fig. 2c), in contrast to the condition seen in both toothed and beaked vertebrates in which they merely communicate with the mandibular canal.

On the ventral surface of the symphysis there is small flat area, rather than a distinct hourglass-shape or dumbbell-shaped depression in *Caenagnathus* and *Chirostenotes* (Fig. 1e)^5^. This flat area is likely to have played a similar role as the depression which accommodates the genioglossus muscle^5,6,9^. This area has a U-shaped anterior margin, whereas its posterior margin bears two notches (Fig. 1e), from which a pair of troughs on the posterior surface of the symphysis extend posterolaterally into the Meckelian grooves. Between the troughs and on the posterior aspect of the symphyseal shelf, there is a shallow midline groove that extends upward from the ventral margin at a distance about half of the dorsoventral height of the symphyseal shelf (Fig. 1c). In *C*. *martinsoni* CCMGE 401/12457, the posterior surface of the symphysis is relatively smooth^5^, whereas there is a tubercle present in this area in ZIN PH 2354/16^6^. The giant caenagnathid MPC-D 107/17 from Mongolia bears a tab-like process dorsal to the Meckelian groove on each side of the sagittal line^8^, but this region is relatively flat in IVPP V20377 (Fig. 1c).

Lateral to the midline groove, the trough-like Meckelian groove extends dorsolaterally for a short distance from a deep fossa that is located on each side of the sagittal plane close to the ventral margin of the symphyseal shelf when viewed posteriorly. It then bends abruptly posteriorly along the lingual aspect of the bone (Fig. 1c). The anterior portion of the Meckelian groove is much shallower than the posterior portion, and is roofed by the swollen lingual ridge as in ZIN PH 2354/16^6^. This swollen lingual ridge has been shown to be a structure accommodating teeth^17^, which is present only in toothed taxa and taxa with ontogenetic tooth reduction. A small elongate foramen is situated where the Meckelian groove inflects (Fig. 1c). This foramen is also present in ZIN PH 2354/16^6^ but absent in other caenagnathids, it is so inconspicuous that it was ignored by previous authors^3^. On the posterior surface of the symphyseal shelf and immediately lateral to this foramen, there is another oval vascular foramen (Fig. 1c), a feature that is also known in ZIN PH 2354/16^6^. Sues and Averianov suggested both of the foramina are channels for the interior alveolar nerve and the internal mandibular arteries^6^. However, our CT images show the small elongate foramen internally leads to a short blind tube that extends anteromedially until it reaches a position right beneath the posterolateral corner of the anterior occlusal groove (Fig. 1f), leaving only the larger oval foramen leading into the internal hollow space. This evidence suggests the small elongate foramen is likely to have accommodated the anterior extension of the Meckelian cartilage when the animal was alive, and the larger oval foramen is homologous to the one that conducted the mandibular nerves and arteries in other caenagnathids^5^. This configuration of paired foramina on the posterior aspect of the symphyseal region of the dentary has been previously reported only in *Allosaurus* ^24,25^, *Duriavenator* ^26^, *Falcarius* ^27^, *Deinonychus* ^28^, *Dromaeosaurus* ^25^ and *Troodon* ^25^ among other theropods, and its presence in *Caenagnathasia* suggest this feature also characterizes some, but not all caenagnathid oviraptorosaurians. Posterior to this oval foramen, almost the entire lingual surface of the dentary ramus is occupied by a dorsoventrally broad and shallow depression (Fig. 1c), which represents the articular surface for the articular-surangular-coronoid complex as suggested by previous authors^6^. Unlike ZIN PH 2354/16^6^, this facet is not divided into dorsal and ventral parts, representing either an interspecifically or individually variable feature.

## Discussion

IVPP V20377 can be referred to Caenagnathidae, and more specifically *Caenagnathasia*, based on a series of synapomorphies including the fused dentary with broadly concave anterodorsal margin, absence of a downturned anterior portion of the dentary, presence of a lingual triturating shelf, presence of “lateral occlusal ridge” (vestigial interdental septa) between the “lateral occlusal grooves” (vestigial alveoli), and the pneumatic dentary^2,6^. In addition to the previously known individual differences^3^, we demonstrate that the depth of the midline groove on the dorsal surface of the dentary symphyseal region, the foramina on the wall of anterior occlusal groove as well as on the lingual ridges are also individually variable features of *Caenagnathasia*. Comparisons of the internal structures with other *Caenagnathasia* specimens are impossible due to the lack of available 3D reconstruction in these specimens.

Among the diagnostic characters of caenagnathid oviraptorosaurs, the “lateral occlusal grooves” and the “lateral occlusal ridges” inside the symphyseal shelf of the dentary often receive special attention. These features were regarded as the diagnostic features of caenagnathids, although their nature and possible functions have long been controversial^5,7,8^. Our previous work suggests that ontogenetic edentulism is present in caenagnathids, and that the lateral occlusal grooves and ridges are homologous to the vestigial alveoli and interdental septa, respectively^17^. This inference suggests that the presence or absence of vestigial alveoli and interdental septa, and their exact numbers and morphology, can be influenced by ontogeny.

Among oviraptorosaurians, teeth are present in both the upper and lower jaws of the most basal taxa *Insicivosaurus* ^29^, *Protarchaeopteryx* ^30^ and *Ningyuansaurus* ^31^, whereas they are only present in the upper jaw in *Caudipteryx* ^30^. In addition, all known oviraptorids and caenagnathids are edentulous, suggesting that the dentary might have been the first jaw bone that becomes completely edentulous in the evolution of oviraptorosaurs. Because caenagnathids have jaw bones that morphologically and functionally resemble those of the basal oviraptorosaurs in many aspects (e.g. the posteriorly located external mandibular fenestra and the long and low mandible indicate caenagnathids and toothed basal oviraptorosaurs may have bitten in a similar way, the caenagnathids’ beak is clearly not as strong as that of oviraptorids^32^ which is likely to have been strengthened by vestigial teeth), and because the ontogenetic remodeling of the occlusal margin of the jaw bones is not as serious as that seen in *Limusaurus* and *Sapeornis* (e.g., the occlusal margin of dentary grows faster than the ventral margin, leading to the dentary anterior end becoming progressively downturned during ontogeny in *Limusaurus* ^18^), vestigial alveoli and interdental septa produced by the ontogenetic edentulism are likely to be preserved in caenagnathids rather than in other oviraptorosaurs. This inference seems to suggest that teeth should have been present in the embryonic or perinatal caenagnathids, which were then progressively reduced in more mature individuals. In fact, vestigial alveoli and interdental septa are present in all known caenagnathids except *Beibeilong*, a perinatal caenagnathid preserved in associate with very large eggs^19^. This appears to contradict to our hypothesis, but we suggest that the absence of vestigial dentition related structures in *Beibeilong* does not conflict with our previous conclusion that the evolutionary tooth reduction of oviraptorosaurs occurred through heterochronic truncation of odontogenesis^17^.

While previous works have suggested that a tooth reduction trend did exist in theropods on the line to birds^17,33^, complete edentulism occurred independently only in a few lineages, and the reappearance of teeth after complete edentulism is not rare^17,33^. For example, in contrast to complete edentulous condition seen in the oldest known beaked bird *Confucisornis* ^34^, tooth reduction and reappearance have repeated for several times in ornithothoraces^35–37^. The repeated absence and reappearance of teeth is likely to have led by heterochronic truncation of odontogenesis through affecting the onset and offset of tooth development, and tooth replacement frequency, rather than gene mutations^17,18^. From this perspective, there are three alternative hypotheses that could probably explain the absence of vestigial dentition related structures in *Beibeilong*: (A) *Beibeilong* is an edentulous caenagnathid. The absence of teeth in *Beibeilong* and presence of teeth in other caenagnathids just like the disappearance and reappearance of teeth in ornithothoraces. (B) teeth existed, but tooth regression occurs before the eruption of the null generation of teeth such that no vestigial alveoli and interdental septa would be present in any developmental stage of *Beibeilong*. Although neither hypothesis A nor B can be tested at present due to the lack of available CT data of *Beibeilong*, it should be noted that a short ridge is present on the lingual aspect of the dentary in this specimen (Fig. 3c in ref.^19^), which resemble the lingual ridge present in toothed vertebrates though it is not as prominent as that seen in other caenagnathids (e.g. IVPP V20377). This seems to disprove hypothesis A, but we acknowledge that more solid evidences are needed to show whether unerupted teeth are preserved inside the jaw bones of this specimen. (C) tooth eruption was significantly delayed while tooth reduction was accomplished much faster than in *Limusaurus* and *Sapeornis*. Because the null-generation teeth of reptiles usually erupts early during embryonic development^38–40^, this hypothesis predicts that teeth, once they erupted in *Beibeilong*, must have been delayed longer than in most other reptiles including crocodilians and most other toothed dinosaurs^39,40^, and disappeared shortly after the eruption. Because CT image could only determine whether unerupted teeth are preserved inside the jaw bones but could not discriminate erupting teeth from the regressing ones, this hypothesis could be proved only when vestigial alveoli are found in more mature *Beibeilong*.

It should be noted that the vestigial alveoli and interdental septa are confined to the anterior portion of the dentary in caenagnathids, and they are especially circular anteriorly (e.g. CCMGE 401/12457 and IVPP V20377) whereas they become transversely constricted and shallow posteriorly. This differs from the tooth reduction pattern seen in *Limusaurus* in which the tooth loss initiates from both the anterior end of the dentary and the mid-portion of the tooth row^18^. In addition, the subcircular vestigial alveoli connect with the hollow interior of the dentary in the relatively small specimens of caenagnathid (e.g. IVPP V20377), whereas in relatively large caenagnathids (CMN 8776^23^, CM 78000^2^, MPC-D 107/17^8^), they are enclosed semicircular to subcircular fossae with the vestigial interdental septa only protruding a short distance lingually from the occlusal edge^4,5^. This suggests that the dentary of large caenagnathids might have experienced more significant remodeling than that of the small specimens, and the remodeling of the interdental septa starts from the lingual side in caenagnathid oviraptorosaurians, which produced a groove next to the lingual wall of the alveoli that morphologically resembles the paradental groove seen in some other theropods^25,41–43^. Previous authors have suggested that the paradental groove is likely a structure that accommodated blood vessels^25^ or dental lamina^44^ when the animal was alive. However, in extant toothed amniotes, the dental lamina is usually present inside the jaw bones and situates lingually close to the functional tooth root^45,46^, and there is no example in extant animals showing a dental lamina placed as high as the dorsal margin of the alveoli. The new information from caenagnathids suggests the remodeling of the interdental septa could result in a groove labial to the lingual wall of the alveoli, which is likely to have accommodated the blood vessels that nourished the rhamphotheca, or merely strengthened the rhamphotheca. This condition is different from *Limusaurus* ^18^, in which there is no sign of vestigial interdental septa nor vestigial alveoli present on the occlusal surface of the jaw bones.

The different jaw bone remodeling patterns are likely to have been correlated with various tooth reduction patterns, and may have produced different occlusal areas. In *Limusaurus*, the anterior dentary teeth lost early during ontogeny, and the anterior end of the dentary becomes downturned after tooth reduction^18^. In contrast, the anterior end of the dentary of *Sapeornis* exhibits little morphological change during ontogeny^17^, which is probably due to the retention of the anterior dentary teeth in this taxon. Although currently there is no available growth series of caenagnathids, their anterior dentary teeth lost early during ontogeny but the anterior end of the dentary exhibits little downturn if compare large specimens to the smaller closely related species. The confluent transversely narrow jaw bones seen in *Limusaurus* suggest occlusion between the upper and lower jaws is limited in this taxon, whereas the retention of the vestigial alveoli in caenagnathids may suggest a transversely wider occlusal surface similar to the condition seen in many turtles^47^. From this perspective, the condition seen in *Sapeornis* is probably intermediate between the conditions of *Limusaurus* and caenagnathids, in which the vestigial teeth remain at the rostral end of the dentary while lost posteriorly^17^, suggesting its limited occlusion is only confined to the rostral end of the jaws and possibly have been strengthened by the remaining teeth. Although currently no geochemical evidence suggest an ontogenetic dietary shift did exist in caenagnathids and *Sapeornis*, these lines of evidence suggest the ontogenetic dietary shift, if once presented in caenagnathids and *Sapeornis*, may have been different from the condition seen in *Limusaurus*.

In addition to feeding, the beak of extant birds has many functions that teeth do not have, including heat exchange^48^, courtship and sensation^49^. Therefore, it is not difficult to understand why there are many neurovascular foramina present on the lateral surface of the jaw bones of beaked animals. Previous works suggested that in both extant and fossil beaked animals, the neurovascular foramina on the lateral surface of the jaw bones communicate internally with the mandibular canal^17,18^, which would have accommodated branches of nerves and blood vessels, and thus the beaks are enriched in blood vessels and nerves (Figs 1 and 2, white tubes). In contrast, in toothed animals including immature *Limusaurus* ^18^, the neurovascular foramina on the lateral surface of the jaw bones are significantly reduced indicating the interior blood vessels and nerves are not as abundant as those present in beaked animals (Fig. 2). In mature *Limusaurus*, along with caenagnathids and *Sapeornis* in which the ontogenetic edentulism was supposed to have been present^17^, the neurovascular foramina communicate with the mandibular canal through the vestigial alveolar canal (Fig. 2), suggesting at least some of the vestigial alveoli were modified into structures accommodating blood vessels and nerves. Because the rhamphotheca has been suggested to have formed through a peramorphic process in the dinosaur-birds lineage, it is unlikely that neurovascular tubes that later nourish the rhamphotheca, existed prior to tooth reduction (see juvenile *Limusaurus*, Fig. 1 in refs^17,18^). Therefore, most of the interior neurovascular tubes are likely to have formed during the remodeling of the jaw bones in these taxa. If this is true, it would be easy to interpret why caenagnathids have jaw bones that are highly pneumatized, and why some neurovascular foramina, including the pneumatopores present on the lateral surface of the dentary, connect with the hollow interior space in these animals. Based on this evidence, we suggest that the presence of the alveolar canal in mature specimens of these taxa is a functional compensation for the insufficient blood supply in toothed jaw bones, which would have been functionally taken over by the blood vessels branching from the mandibular canal in the later evolution of beak.

## Conclusions

We reevaluated the morphological features of caenagnathid dentaries based on CT images of *Caenagnathiasia* sp. IVPP V20377, and discussed the possible function of the alveolar canal. Based on the new information, we conclude that (1) several previously unknown features, such as the paired fossae on the posterior aspect of the dentary symphyseal region, the morphology of the dentary hollow interior space, the crateriform vestigial alveoli, the paired blind tubes beneath the symphyseal shelf are recognized; (2) the different numbers of vestigial alveoli and interdental septa seen in different caenagnathids could be influenced by patterns of tooth reduction and jaw bones remodeling; (3) the different occlusal area and jaw bone morphologies indicates if the ontogenetic dietary shift once presented in caenagnathids and *Sapeornis*, it may have been different from that seen in *Limusaurus*; (4) as tooth reduction progresses, the alveoli are modified into structures accommodating the blood vessels that nourish the rhamphotheca.

## Material and Methods

IVPP V20377, an isolated symphyseal region of fused dentaries was recovered from the Upper Cretaceous Iren Dabasu Formation (Campanian), Inner Mongolia, China (Fig. 1)^3^. The stratigraphic information and the paleoenvironmental condition of this locality has been reported by previous authors^3,50^.

IVPP V15923 was scanned using SR-μCT (Shanghai Synchrotron Radiation Facility, SSRF)^18^, whereas the rest of specimens were scanned using the Mi-CT 225 kV micro-computerized tomography (developed by the Institute of High Energy Physics, Chinese Academy of Sciences) CT at the Key Laboratory of Vertebrate Evolution and Human Origins, Institute of Vertebrate Paleontology and Paleoanthropology, Chinese Academy of Sciences. All of the specimens were scanned along the sagittal axis, for other testing conditions refer to Wang *et al*.^17^.
